# Protective effect of inhaled corticosteroid on children with asthma with *Mycoplasma pneumoniae* pneumonia

**DOI:** 10.3389/fped.2022.908857

**Published:** 2022-08-25

**Authors:** Bing Wei, Yan-Hong Dang, Xiang-Ping Liu, Miao Li

**Affiliations:** ^1^Department of Neonatology, General Hospital of Northern Theater Command, Shenyang, China; ^2^Department of Pediatrics, Shengjing Hospital of China Medical University, Shenyang, China

**Keywords:** asthma, children, *Mycoplasma pneumoniae* pneumonia, inhaled corticosteroids, exacerbation

## Abstract

**Background:**

The aim of this study was to determine the differences in the characteristics of *Mycoplasma pneumoniae* pneumonia (MPP) in children with and without asthma and in children with asthma with and without inhaled corticosteroid (ICS) therapy in order to determine the risk factors for asthma exacerbation and the effect of regular ICS therapy on children with asthma with MPP.

**Materials and methods:**

Children with MPP were divided into two groups according to whether they had a history of asthma. Children with asthma were further divided into an ICS therapy group and a group without ICS therapy. The clinical characteristics, laboratory test results, and pulmonary images were compared between the children with and without asthma. Differences in the severity of acute exacerbation were compared between the children with asthma in the ICS therapy and without ICS therapy groups. Multivariable logistic regression was used to determine the risk factors for exacerbation of MPP in children with asthma.

**Results:**

In children with MPP, the differences in the eosinophil counts; total immunoglobulin E (IgE), C-reactive protein, procalcitonin (PCT), and lactate dehydrogenase (LDH) levels; and fever duration, wheezing, extrapulmonary complications, oxygen saturation < 92%, severe pneumonia, pleural effusion, co-infection with other pathogens, and lobar pneumonia between children with and without asthma were statistically significant. Among children with asthma with MPP, those in the ICS therapy group were less likely to experience an exacerbation, and exacerbations were less severe than those in the without ICS therapy group. The multivariable logistic regression analysis showed that the ICS therapy was an independent protective factor against exacerbation.

**Conclusion:**

Among children with MPP, the chance of wheezing was higher in children with asthma than in children without asthma. The ICS therapy was a protective factor against exacerbation in children with asthma with MPP.

## Introduction

Asthma is the most common chronic airway disease in children. The main clinical manifestations are repeated wheezing, cough, shortness of breath, and chest tightness, often accompanied by reversible airflow restriction. Children with asthma are often hospitalized for exacerbations, which increases family costs (1). Children with asthma also suffer from prolonged illness due to recurrence, with decreased lung function (2), which affects the quality of life (3). Therefore, it is particularly important to use a standardized approach for treating children with asthma. Clinicians recommend inhaled corticosteroid (ICS) therapy for children with asthma, followed by the guidelines of the Global Initiative for Asthma (GINA) or other regional guidelines to prevent asthmatic attacks (4, 5). Low-dose ICS therapy is more effective than intermittent therapy in preschool children with asthma (6) and is effective at preventing death from asthma (7). ICS can reduce the frequency of rehospitalizations and recurrent respiratory infections in children with asthma (8), the cost of hospitalization, and the dosage of oral glucocorticoids required (9). A meta-analysis has found that ICS therapy can reduce the risk of requiring intravenous glucocorticoid therapy for acute episodes by 17% (10). Randomized controlled trials have shown that long-term ICS therapy significantly reduces the risk associated with oral glucocorticoid therapy for treating the exacerbations of asthma (11). Several studies have focused on the relationship between infection and exacerbation of asthma (12–14) and the underlying mechanisms. However, the effect of ICS on the clinical characteristics of exacerbations in children with asthma and the effect of ICS therapy on children with asthma after the infection have not been reported.

*Mycoplasma pneumoniae* (MP) is one of the most common pathogens of lower respiratory tract infections in children (15, 16). It is also a common cause of asthma exacerbation (17, 18). MP infection is associated with refractory asthma and can lead to decreased lung function (19, 20). Several studies have focused on the mechanism underlying exacerbation of asthma after MP infection, but few studies have investigated the effect of ICS on the clinical characteristics of children with asthma after MP infection. Therefore, this study has investigated the differences in clinical characteristics, laboratory test results, and pulmonary imaging findings in children with MP pneumonia (MPP) with and without asthma, and children with asthma with and without ICS therapy, in order to determine the risk factors for exacerbation and the effectiveness of ICS in preventing exacerbation in children with asthma with MPP.

## Materials and methods

### Participants and inclusion and exclusion criteria

We retrospectively analyzed the medical records of children hospitalized with MPP in the pediatric pulmonary department of Shengjing Hospital in Shenyang, Liaoning, China, from June 2016 to December 2021. Among children with more than one hospital admission for MPP during the study period, only the data from the first admission were included in the analysis. Children with congenital pulmonary dysplasia, airway malformation, congenital immune deficiency disease, congenital heart disease, malnutrition, bronchiolitis obliterans, bronchial foreign bodies, or congenital metabolic diseases were excluded. The requirement for informed consent was waived by the ethics committee. The differences in the basic characteristics, clinical characteristics, laboratory test results, and pulmonary imaging findings between the children with MPP with and without asthma, and between children with asthma with and without ICS therapy were analyzed. Multivariable logistic regression analysis was used to identify the risk factors for exacerbation and to assess the effect of ICS on children with asthma with MPP.

### Study definitions

*Mycoplasma pneumoniae* infection was diagnosed based on positive MP-immunoglobulin M (IgM) antibody test results detected by serological testing and a positive MP-DNA polymerase chain reaction test result of a nasopharyngeal swab sample. Patients with only one positive result were excluded. MP IgM results with a signal-to-cutoff (S/CO) ratio of > 1.1 were defined as positive, those with a S/CO of 0.8–1.1 were defined as weak positive, and those with a S/CO of < 0.8 were defined as negative. MP antibody (IgM antibody kit; Shenzhen PuRuiKang Biological Technology, Co., Ltd., Shenzhen, China) and MP-DNA screening and identification (MP-DNA detection kit; Shenzhen PuRuiKang Biological Technology, Co., Ltd., Shenzhen, China) were performed within 24 h after admission. Pneumonia was diagnosed based on the patient’s symptoms (fever, cough, and/or rapid breathing) and clinical evidence of pneumonia (tachypnea, chest recession, and/or adventitious sounds on lung auscultation) with radiographic signs (infiltrates or consolidation). Severe pneumonia was defined as pneumonia requiring either invasive or non-invasive respiratory support, pediatric intensive care unit care, or when the illness resulted in death. The criteria for asthma diagnosis and the severity of exacerbation were determined according to the GINA 2016 guidelines for the diagnosis and treatment of asthma (4). Asthma exacerbations are episodes characterized by a progressive increase in symptoms of shortness of breath, cough, wheezing, or chest tightness and a progressive decrease in lung function (4).

### Data collection

The clinical records of the children were retrospectively reviewed and the following data were collected: age, sex, fever duration, length of hospital stay, cases of wheezing, severe pneumonia, fibrobronchoscopic treatment, oxygen saturation < 92%, extrapulmonary complications (e.g., myocarditis, hepatitis, encephalitis, thrombocytopenic purpura, autoimmune hemolysis, and skin and mucosal damage), pleural effusion, mechanical ventilation, and co-infection with other pathogens (e.g., *Chlamydia*, adenoviruses, rhinoviruses, or respiratory syncytial virus). Laboratory tests on admission included the neutrophil count, eosinophil count, lymphocyte count, and the levels of C-reactive protein (CRP), procalcitonin (PCT), alanine aminotransferase (ALT), lactate dehydrogenase (LDH), and total immunoglobulin E (IgE). Imaging included chest computed tomography (CT) or X-ray. Children with wheezing required a high-resolution CT (HRCT) scan to exclude abnormal airway development and to observe the small airway changes. Small airway lesions, involvement of multiple lobes, and lobar pulmonary on chest HRCT scan were recorded. Two chest radiography specialists independently reviewed the images.

### Treatment modalities

The diagnosis and treatment of MPP were performed according to the community guidelines for acquired pneumonia issued by the State Administration of the National Health Commission of the People’s Republic of China and national guidelines published in 2019 (21, 22). All the children received standard treatment for MPP, which included supportive care, invasive or non-invasive ventilation, and macrolide antibiotics, as necessary. Intravenous immunoglobulin was administered to children with persistent fever, and methylprednisolone was administered to children with severe disease causing a rapid increase in respiratory distress, at the discretion of the treating physician. Children with wheezing were treated with a bronchodilator to relax the airway. Children with MPP with fever duration of less than 48 h, without wheezing, with relieved cough, and a decrease in infiltrates or the consolidation area on chest imaging after admission were discharged from the hospital. Children with asthma had regular follow-up visits scheduled.

### Patient selection

**FIGURE 1:**
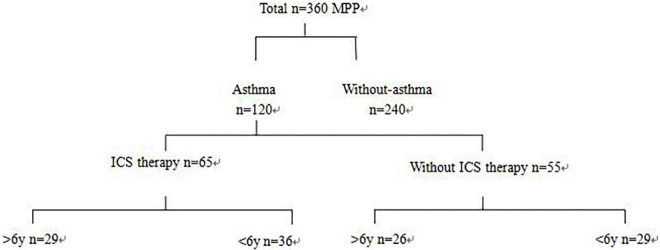
Chart for patient grouping. Three hundred sixty children hospitalized for MPP were retrospectively analyzed. Based on the history of asthma, 120 hospitalized MPP children with asthma were randomly selected, and 240 MPP children without asthma were selected as control. According to the history of treatment, children with asthma with MPP were further divided into two groups: ICS therapy and without ICS therapy groups.

Based on the history of asthma, 120 children with asthma hospitalized with MPP were randomly selected, and 240 children with MPP without asthma were selected as controls. According to their history of treatment, the children with MPP and asthma were further divided into two groups, namely, with ICS therapy (more than 3 months) and without ICS therapy groups (Figure 1). ICS therapy was defined according to the GINA guidelines. Patients were required to have regular follow-up consultations and a follow-up within 3 months after therapy modification until discontinuation (4).

### Statistical analysis

Normally distributed continuous variables were summarized using means ± standard deviations, and independent samples *t*-tests were used to assess the significance of differences between groups. Non-normally distributed continuous variables were summarized as medians and interquartile ranges (IQRs), and the rank-sum test was used to assess the significance of differences between groups. ANOVA was used for counting data, and the mean rank test was used if the theoretical frequency was < 5 or the total number of cases was < 40. The Chi-square test was used to assess the significance of differences in categorical variables between groups, and multivariable logistic regression analysis was used to identify the risk factors associated with acute asthma attacks in children with MPP. As the criteria for diagnosis and treatment of children with asthma aged less than and over 6 years are different (4, 23), we analyzed the characteristics separately. All statistical analyses were conducted using SPSS 20.0 (IBM Corp., Armonk, NY, United States). Two-sided *P*-values < 0.05 were considered statistically significant.

## Results

The records of 360 children aged 1–14 years, hospitalized for MPP from June 2016 to December 2021, were included in the analysis.

### Baseline characteristics of children with *Mycoplasma pneumoniae* pneumonia with and without asthma

There were no statistically significant differences in sex and age between the children with MPP with and without asthma (Table 1).

**TABLE 1:** Baseline characteristics of children with MPP with and without asthma.

Variable	Without-asthma (*n* = 240) *n* (%)	Asthma (*n* = 120) *n* (%)	X^2^	*P*-value
Sex			3.472	0.062
Male *n* (%)	111 (46.25%)	68 (56.67%)		
Female *n* (%)	129 (53.75%)	52 (43.33%)		
Age			1.693	0.193
≥ 6 years *n* (%)	87 (36.25%)	52 (43.33%)		
< 6 years *n* (%)	153 (63.75%)	68 (56.67%)		

### Clinical characteristics of children with *Mycoplasma pneumoniae* pneumonia with and without asthma

There were statistically significant differences in the eosinophil counts, total IgE, and CRP, PCT, and LDH levels between the children with MPP with and without asthma. There were no significant differences in the white blood cell count, neutrophil count, monocyte count, or ALT levels between the two groups. There were statistically significant differences between the two groups in the duration of fever and incidence of wheezing, extrapulmonary complications, oxygen saturation < 92%, severe pneumonia, pleural effusion, and co-infection with other pathogens. There were no statistically significant differences in the length of hospital stay, the incidence of severe cough, or the need for mechanical ventilation between the two groups. There was a significant difference in the incidence of lobar pneumonia, but there were no statistically significant differences in the incidence of small airway changes and multilobar involvement in pulmonary imaging between the two groups (Table 2).

**TABLE 2:** Clinical characteristics of the children with MPP with and without asthma.

Variable	Without-asthma (*n* = 240) *n* (%)	Asthma (*n* = 120) *n* (%)	Z/X^2^	*P*-value
**Laboratory test**				
White blood cell count (×10^9^/L)	8.48 (5.81,11.18)	9.19 (6.64,12.08)	–1.892	0.058
Neutrophil count (×10^9^/L)	4.90 (3.10,7.40)	5.60 (3.13,9.28)	–1.777	0.076
Leukomonocyte count (×10^9^/L)	2.20 (1.60,2.90)	2.15 (1.50,3.20)	–0.008	0.994
Eosinophils count (×10^9^/L)	0.06 (0.01,0.19)	0.20 (0.04,0.40)	–5.536	0.010*
Total IgE (IU/mL)	74.51 (28.34,181.15)	186.30 (61.25,575.85)	–4.012	< 0.001*
CRP (mg/L)	14.10 (4.69,29.85)	7.30 (2.47,14.58)	4.511	< 0.001*
PCT (ng/mL)	0.10 (0.06,0.24)	0.07 (0.05,0.12)	4.363	< 0.001*
LDH (U/L)	276.00 (245.50,327.50)	240.00 (218.50,277.75)	5.350	< 0.001*
ALT (U/L)	13.00 (10.00,19.75)	12.00 (10.00,19.00)	0.717	0.473
**Clinical characteristics**				
length of hospital stay (day)	8.00 (6.00,9.00)	7.00 (6.00,9.00)	1.501	0.133
Fever duration (day)	8.00 (5.00,10.00)	4.50 (2.00,7.00)	5.415	< 0.001*
Wheezing *n* (%)			215.77	< 0.001*
Y	23 (9.58%)	106 (88.33%)		
N	217 (90.42%)	14 (11.67%)		
Extrapulmonary complications *n* (%)			6.197	0.013*
Y	20 (8.33%)	2 (1.67%)		
N	220 (91.67%)	118 (98.33%)		
Oxygen saturation < 92% *n* (%)			31.250	< 0.001*
Y	9 (3.75%)	27 (22.50%)		
N	231 (96.25%)	93 (77.50%)		
Severe pneumonia *n* (%)			5.863	0.015*
Y	73 (12.08%)	40 (37.74%)		
N	167 (69.58%)	66 (62.26%)		
Pleural effusion *n* (%)			8.371	0.004*
Y	31 (12.92%)	4 (3.33%)		
N	209 (87.08%)	116 (96.67%)		
Need for Mechanical ventilation *n* (%)			1.934	0.164
Y	3 (1.25%)	5 (4.17%)		
N	237 (98.75%)	115 (95.83%)		
Co-infection with other pathogens *n* (%)			10.655	0.001*
Y	100 (41.67%)	29 (24.17%)		
N	140 (58.33%)	91 (75.83%)		
**Pulmonary images**				
Small airway changes *n* (%)			0.33	0.566
Y	7 (2.92%)	8 (6.67%)		
N	233 (97.08%)	112 (93.33%)		
Multilobe involvement (≥ 3) *n* (%)			0.632	0.426
Y	30 (12.50%)	21 (17.50%)		
N	210 (87.50%)	99 (82.50%)		
Lobar pulmonary *n* (%)			24.863	< 0.001*
Y	36 (15.00%)	4 (3.33%)		
N	204 (85.00%)	116 (96.67%)		

*P < 0.05, compare with children with MPP with asthma statistically significant. CRP, C-reactive protein; PCT, procalcitonin; LDH, lactate dehydrogenase; ALT, alanine transaminase.

### Risk factors for asthma exacerbation in children with asthma with *Mycoplasma pneumoniae* pneumonia

Many children with asthma with MPP experienced acute episodes of asthma. The basic characteristics, clinical characteristics, laboratory test results, and pulmonary imaging findings of children with asthma with MPP with and without asthma exacerbation are shown in Table 3. Multivariable logistic regression analysis revealed that allergen exposure, severe pneumonia, co-infection with other pathogens, and higher total IgE levels were not associated with asthma exacerbation in children with asthma with MPP. However, ICS therapy was found to be a protective factor against asthma exacerbation in children with asthma [odds ratio (OR): 0.167] (Table 4).

**TABLE 3:** Basic and clinical characteristics of the children with MPP with asthma exacerbation.

Variable	Exacerbation (*n* = 106)	Without exacerbation (*n* = 14)	t/χ^2^/z	*P*
Sex			0.459	0.498
Male *n* (%)	58	9		
Female *n* (%)	48	5		
Age			0.137	0.711
≥ 6 years *n* (%)	51	6		
< 6 years *n* (%)	55	8		
ICS therapy *n* (%)	53 (50%)	12 (85.71%)	6.354	0.012*
Co-infection with other pathogens *n* (%)	22 (20.75%)	7 (50%)	4.286	0.038*
Allergen exposure *n* (%)	38 (35.85%)	9 (64.29%)	4.197	0.040*
Small airway changes *n* (%)	7 (6.60%)	1 (7.14%)	–	1.000
Total IgE	214.50 (68.79,576.00)	64.37 (28.21,189.78)	2.184	0.029*
Eosinophils count (×10^9^/L)	0.20 (0.04,0.40)	0.18 (0.04,0.42)	0.200	0.841
Severe pneumonia *n* (%)	43 (40.57)	3 (21.43%)	1.916	0.166
CRP	7.13 (2.57,13.70)	14.42 (2.00,24.13)	0.764	0.445

*P < 0.05, compare with children with MPP without asthma exacerbation statistically significant.

**TABLE 4:** Risk factors for exacerbation to MPP children with asthma by multivariate logistic analysis.

Variable	Exacerbation (*n* = 106) *n* (%)	Without exacerbation (*n* = 14) *n* (%)	X^2^	*P*-value	Odds ratio 95% (confidence interval)
ICS therapy *n* (%)	53 (50%)	12 (85.71)	5.169	0.023*	0.167 (0.036,0.781)
Total IgE (IU/mL)	432.26 ± 562.06	116.16 ± 206.56		0.040	1.005 (1.000–1.010)
Allergen explosure *n* (%)	38 (35.85%)	9 (64.29%)	4.197	0.286	0.377 (0.063–2.264)
Co-infection with other pathogens *n* (%)	22 (20.75%)	7 (50%)	2.700	0.298	0.404 (0.073–2.229)

*P < 0.05, compare with without exacerbation group statistically significant.

### Differences in clinical characteristics between the children with asthma with and without inhaled corticosteroid therapy after *Mycoplasma pneumoniae* pneumonia

The differences in clinical characteristics, laboratory tests, and pulmonary images between children with asthma with and without ICS therapy after MPP are shown in Table 5. There were statistically significant differences in the incidence of asthma exacerbation and fever duration, but there were no statistically significant differences in the laboratory test results or pulmonary imaging findings between the two groups.

**TABLE 5:** Clinical characteristics of children with MPP with asthma.

Variable	Without ICS therapy (*n* = 55) *n* (%)	ICS therapy (*n* = 65) *n* (%)	Z/X^2^	*P*-value
**Laboratory test**				
White blood cell count (×10^9^/L)	9.72 ± 3.79	9.20 (6.70,12.15)	0.040	0.968
Neutrophil count (×10^9^/L)	4.30 (2.60,9.20)	6.00 (3.65,9.30)	–1.18	0.238
Leukomonocyte count (×10^9^/L)	2.00 (1.30,3.60)	2.30 (1.60,2.95)	–0.179	0.858
Eosinophils count (×10^9^/L)	0.21 (0.04,0.40)	0.20 (0.04,0.40)	0.622	0.534
Total IgE (IU/mL)	203.65 (54.64,562.50)	186.00 (59.77,579.70)	0.095	0.924
CRP (mg/L)	6.91 (2.39,13.70)	8.70 (2.54,17.10)	–0.737	0.461
PCT (ng/mL)	0.07 (0.05,0.11)	0.08 (0.05,0.16)	–0.404	0.687
LDH (U/L)	246.00 (219.00,289.50)	239.00 (218.00,277.00)	–0.461	0.645
ALT (U/L)	12.00 (9.00,19.00)	13.00 (10.00,20.00)	–1.098	0.272
**Clinical characteristics**				
length of hospital stay (day)	7.00 (6.00,8.00)	7.00 (6.00,9.00)	–1.400	0.162
Fever duration (day)	4.00 (2.00,6.00)	5.00 (3.00,8.00)	–1.980	0.048*
Cases of Exacerbation *n* (%)			6.354	0.012*
Y	53 (96.36%)	53 (81.54%)		
N	2 (3.63%)	12 (18.46)		
Extrapulmonary complications *n* (%)			−	0.208
Y	2 (3.64%)	0 (0%)		
N	53 (96.36%)	65 (100%)		
Oxygen saturation < 92% *n* (%)			1.326	0.249
Y	15 (27.27%)	12 (18.46%)		
N	40 (72.73%)	53 (81.54%)		
Severe pneumoniae *n* (%)			0.017	0.897
Y	18 (32.73%)	22 (33.85%)		
N	37 (67.27%)	43 (66.15%)		
Pleural effusion *n* (%)			0.000	1.000
Y	2 (3.64%)	2 (3.08%)		
N	53 (96.36%)	63 (96.92%)		
Need for Mechanical ventilation *n* (%)			0.036	0.849
Y	3 (5.45%)	2 (3.08%)		
N	52 (94.55%)	63 (96.92%)		
Co-infection with other pathogens *n* (%)			3.373	0.066
Y	9 (16.36%)	20 (30.77%)		
N	46 (83.64%)	45 (69.23%)		
**Pulmonary images**				
Small airway changes *n* (%)			0.015	0.903
Y	3 (5.45%)	5 (7.69%)		
N	52 (94.55%)	60 (92.31%)		
Multilobe involvement (≥ 3) *n* (%)			0.614	0.433
Y	8 (14.55%)	13 (20%)		
N	47 (85.45%)	52 (80%)		
Lobar pulmonary *n* (%)			0.116	0.734
Y	1 (1.82%)	3 (4.62%)		
N	54 (98.18%)	62 (95.38%)		

*P < 0.05, compare with ICS therapy group statistically significant.

### Differences in the severity of asthma exacerbation between the children with asthma with and without inhaled corticosteroid therapy after *Mycoplasma pneumoniae* pneumonia

The children aged less than 6 years (36 children in the ICS therapy group and 29 children in the without ICS therapy group) were divided into three groups (without attack, mild, and severe degree). The children aged above 6 years with acute exacerbation (29 children in the ICS therapy group and 26 children in the without ICS therapy group) were divided into five groups (without attack, mild, moderate, severe, and life-threatening). Among children aged less than 6 years, the severity of exacerbation in the ICS therapy group was milder than those in the without ICS therapy group, but there was no significant difference between the two groups among children aged more than 6 years (Table 6).

**TABLE 6:** Severity of asthma exacerbation for MPP children with asthma.

Age	Group	Without exacerbation	Mild	Moderate	Severe	Life-threatening	Mean Rank	Rank Sum	Z	*P*
≥ 6 years	ICS therapy group (*n* = 29) *n* (%)	4 (13.8%)	7 (24.1%)	6 (20.7%)	11 (37.9%)	1 (3.4%)	27.86	808	−0.071	0.944
	Without ICS therapy group (*n* = 26) *n* (%)	2 (5.6%)	9 (25.0%)	4 (11.1%)	10 (38.5%)	1 (2.8%)	28.15	732		
< 6 years	ICS therapy group (*n* = 36) *n* (%)	8 (22.2%)	13 (36.1%)		15 (41.7%)		28.82	1037.5	−2.197	0.028*
	Without ICS therapy group (*n* = 29) *n* (%)	0 (0%)	11 (37.9%)		18 (62.1%)		38.19	1107.5		

*P < 0.05, compare with the children in without ICS therapy group statistically significant.

## Discussion

Bronchial asthma is a chronic inflammatory airway disease, which is usually characterized by bronchospasm and airway hyperresponsiveness. Various pathogens can cause damage to the airway epithelium. MP is a common pathogen that causes respiratory tract infections in children. It can induce various inflammatory cells to produce inflammatory factors [e.g., interleukin 4 (IL-4), IL-13, CC motif chemokine ligand 17 (CCL17), CCL22, and transforming growth factor-beta (TGF-β)], and can increase airway hyperreactivity (24). The P1 and HMW1 proteins secreted by MP can help the pathogen adhere to airway epithelial cells and resist cilial clearance (25). The hydrogen peroxide and CARDS TX secreted by MP can damage the airway and induce large inflammatory cell infiltrations, leading to lung consolidation (26).

In this study, we compared the differences in laboratory test results, clinical characteristics, and pulmonary imaging of children with MPP with and without asthma. There were significant differences in the eosinophil counts and total IgE levels between the children with MPP with and without asthma. The reason for the difference may be related to the allergen exposure of children with asthma, which is unrelated to the MPP. Therefore, allergen exposure avoidance is important for children with asthma. In this study, we also found that the levels of CRP, ALT, and PCT and the incidence of wheezing, extrapulmonary complications, oxygen saturation < 92%, severe pneumonia, pleural effusion, and lobar pulmonary perfusion of the children with MPP and asthma were lower than those of the children with MPP without asthma. These clinical features are indicators of serious MPP, which suggests that the severity of children with asthma with MPP is milder than that of children without asthma with MPP. This may be due to the use of glucocorticoids to treat acute attack. To the best of our knowledge, no previous studies have reported on the clinical characteristics of children with asthma with MPP.

Inhaled corticosteroid is the first-choice treatment for bronchial asthma. It can maintain a certain concentration of glucocorticoids in the airway, causing a certain inhibitory effect on airway hyperresponsiveness, and can reduce the dosage of glucocorticoids required during exacerbations. Compared with oral and systemic corticosteroids, ICS can directly reach the site with lower side effects and work synergistically with systemic glucocorticoids in patients with life-threatening diseases (27). Many children with asthma in this study experienced exacerbations after MPP, but some children with asthma did not. ICS therapy prevented exacerbations in children with asthma with MPP. However, allergen exposure and co-infection were risk factors for exacerbation in children with asthma. Therefore, long-term treatment of ICS in children with asthma can reduce the severity of acute attacks, and exposure to allergens should be avoided. Antibiotic use (28) and family history of asthma (29) are risk factors for recurrent hospitalization for acute attacks in children with asthma. Children with asthma require chronic treatment and medium- and high-dose ICS should be used in the acute stage and low-dose ICS should be used in the remission stage after the acute stage. Treatment should be continued for 3 months before deciding whether to reduce the dosage. Therefore, in this study, we selected patients with asthma who had been on ICS therapy for more than 3 months in order to avoid the bias of results caused by the difference in the dose of ICS. We divided children with asthma with MPP into two groups, namely, ICS therapy and without ICS therapy groups, according to whether they were treated with ICS for 3 months after asthma diagnosis. There were significant differences in the incidence of asthma exacerbation and fever duration, but there were no differences in the laboratory test results and pulmonary imaging findings among children with asthma with and without ICS therapy. The severity of the exacerbation in the ICS therapy group was milder than that in the without ICS therapy group of children aged less than 6 years. There were also significant differences in the fever duration and incidence of wheezing between the groups. The reason for the difference in fever duration may be because glucocorticoid use shortened the fever duration.

We analyzed the differences in the severity of asthma exacerbation between the children with asthma with and without ICS therapy after MPP. Among children with asthma aged less than 6 years, the exacerbations in the ICS therapy group were less severe than those in the without ICS therapy group, but there was no significant difference between the two groups of children aged more than 6 years. This suggests that ICS therapy after asthma diagnosis can reduce the severity of exacerbation in children with asthma aged less than 6 years. The onset of asthma before the age of 6 years is an important risk factor (30), but the effect of ICS therapy after asthma diagnosis on children with asthma exacerbation has not been reported previously. Among children with asthma, long-term wellness control is very important in daily life and for mental health. Therefore, children with asthma should have regular follow-ups scheduled and should take part in self-management according to an asthma action plan (31) to reduce recurrence and maintain asthma wellness control. This can lead to reductions in medical costs related to rehospitalization (32) and side effects of systemic steroid application due to exacerbation.

In summary, in this study, we can conclude that among children with MPP, the chance of wheezing was higher in children with asthma than in children without asthma. ICS therapy was a protective factor against exacerbation in children with asthma with MPP. This study has some limitations. First, it was a retrospective study. There are no studies to date on the long-term outcomes of children with asthma with MPP; therefore, we decided to assess the frequency of acute episodes and the impact of MPP on children with asthma after they had been discharged. Differences in the education level of the children’s guardians may affect their knowledge of diseases, which may influence the choice of treatment methods for children with asthma before hospitalization with acute episodes (33), such as the inhaled drug choice. This may also have influenced the hospitalization rate.

## Data availability statement

The original contributions presented in this study are included in the article/supplementary material, further inquiries can be directed to the corresponding author.

## Author contributions

ML conceptualized and designed the study, drafted the initial manuscript, and reviewed and revised the manuscript. X-PL and Y-HD designed the data collection instruments, coordinated and supervised data collection, and reviewed and revised the manuscript. BW performed the initial analyses and critically reviewed the manuscript for important intellectual content. All authors approved the final manuscript as submitted and agreed to be accountable for all aspects of the study.

## Abbreviations

MP: *Mycoplasma pneumoniae*
MPP: *Mycoplasma pneumoniae* pneumonia
ICS: inhaled corticosteroid
CRP: C-reactive protein
ALT: alanine aminotransferase
GINA: Global Initiative for Asthma
PCT: procalcitonin
LDH: lactate dehydrogenase
CT: computed tomography
HRCT: high-resolution computed tomography
IgE/M: immunoglobulin E/M.

## References

[1] Pugliese FR Guglielmelli E Angelini D Cicchini C Castaldo E Di Girolamo F Pharmacoeconomic management of patient with severe asthma in the emergency department: retrospective multicentric and cost of illness study. *Eur Rev Med Pharmacol Sci.* (2020) 24:11729–39. 10.26355/eurrev_202011_23824 33275242

[2] Tenero L Zaffanello M Piazza M Piacentini G. Measuring airway inflammation in asthmatic children. *Front Pediatr.* (2018) 6:196. 10.3389/fped.2018.00196 30035104 PMC6043865

[3] Serebrisky D Wiznia A. Pediatric asthma: a global epidemic. *Ann Glob Health.* (2019) 85:6. 10.5334/aogh.2416 30741507 PMC7052318

[4] Horak F Doberer D Eber E Horak E Pohl W Riedler J Diagnosis and management of asthma – statement on the 2015 GINA guidelines. *Wien Klin Wochenschr.* (2016) 128:541–54. 10.1007/s00508-016-1019-4 27370268 PMC5010591

[5] Rothe T Spagnolo P Bridevaux PO Clarenbach C Eich-Wanger C Meyer F Diagnosis and management of asthma – the Swiss guidelines. *Respiration.* (2018) 95:364–80. 10.1159/000486797 29614508

[6] Murphy KR Hong JG Wandalsen G Larenas-Linnemann D El Beleidy A Zaytseva OV Nebulized inhaled corticosteroids in asthma treatment in children 5 years or younger: a systematic review and global expert analysis. *J Allergy Clin Immunol Pract.* (2020) 8:1815–27. 10.1016/j.jaip.2020.01.042 32006721

[7] Suissa S Ernst P Benayoun S Baltzan M Cai B. Low-dose inhaled corticosteroids and the prevention of death from asthma. *N Engl J Med.* (2000) 343:332–6. 10.1056/NEJM200008033430504 10922423

[8] Chipps BE Schnepp CM Briscoe M. Budesonide inhalation suspension reduces the need for emergency intervention in pediatric asthma: a named-patient case series. *J Asthma.* (2003) 40:895–900. 10.1081/jas-120023581 14736089

[9] Kew KM Quinn M Quon BS Ducharme FM. Increased versus stable doses of inhaled corticosteroids for exacerbations of chronic asthma in adults and children. *Cochrane Database Syst Rev.* (2016) 2016:CD007524. 10.1002/14651858.CD007524.pub4 27272563 PMC8504985

[10] Nuhoglu Y Atas E Nuhoglu C Iscan M Ozcay S. Acute effect of nebulized budesonide in asthmatic children. *J Investig Allergol Clin Immunol.* (2005) 15:197–200. 16261956

[11] Castro-Rodriguez JA Rodrigo GJ. The role of inhaled corticosteroids and montelukast in children with mild-moderate asthma results of a systematic review with meta-analysis. *Arch Dis Child.* (2010) 95:365–70. 10.1136/adc.2009.169177 19946008

[12] Guibas GV Tsolia M Christodoulou I Stripeli F Sakkou Z Papadopoulos NG. Distinction between rhinovirus-induced acute asthma and asthma-augmented influenza infection. *Clin Exp Allergy.* (2018) 48:536–43. 10.1111/cea.13124 29473978

[13] Itagaki T Aoki Y Matoba Y Tanaka S Ikeda T Mizuta K. Clinical characteristics of children infected with enterovirus D68 in an outpatient clinic and the association with bronchial asthma. *Infect Dis (Lond).* (2018) 50:303–12. 10.1080/23744235.2017.1400176 29119851

[14] Saglani S Fleming L Sonnappa S Bush A. Advances in the aetiology, management, and prevention of acute asthma attacks in children. *Lancet Child Adolesc Health.* (2019) 3:354–64. 10.1016/S2352-4642(19)30025-2 30902628

[15] Kumar S. *Mycoplasma pneumoniae*: a significant but underrated pathogen in paediatric community-acquired lower respiratory tract infections. *Indian J Med Res.* (2018) 147:23–31. 10.4103/ijmr.IJMR_1582_16 29749357 PMC5967212

[16] Kutty PK Jain S Taylor TH Bramley AM Diaz MH Ampofo K *Mycoplasma pneumoniae* among children hospitalized with community-acquired pneumonia. *Clin Infect Dis.* (2019) 68:5–12. 10.1093/cid/ciy419 29788037 PMC6552676

[17] Iramain R De Jesús R Spitters C Jara A Jimenez J Bogado N *Chlamydia pneumoniae*, and *Mycoplasma pneumoniae*: are they related to severe asthma in childhood? *J Asthma.* (2016) 53:618–21. 10.3109/02770903.2015.1116085 27120360

[18] Kumar S Roy RD Sethi GR Saigal SR. *Mycoplasma pneumoniae* infection and asthma in children. *Trop Doct.* (2019) 49:117–9. 10.1177/0049475518816591 30537911

[19] Ansarin K Abedi S Ghotaslou R Soroush MH Ghabili K Chapman KR. Infection with *Mycoplasma pneumoniae* is not related to asthma control, asthma severity, and location of airway obstruction. *Int J Gen Med.* (2010) 4:1–4. 10.2147/IJGM.S15867 21403784 PMC3056323

[20] Shin JE Cheon BR Shim JW Kim DS Jung HL Park MS Increased risk of refractory *Mycoplasma pneumoniae* pneumonia in children with atopic sensitization and asthma. *Korean J Pediatr.* (2014) 57:271–7. 10.3345/kjp.2014.57.6.271 25076972 PMC4115068

[21] Lynch JP III Kajon AE. Adenovirus: epidemiology, global spread of novel serotypes, and advances in treatment and prevention. *Semin Respir Crit Care Med.* (2016) 37:586–602. 10.1055/s-0036-1584923 27486739 PMC7171713

[22] Rogozinski LE Alverson BK Biondi EA. Diagnosis and treatmen of *Mycoplasma pneumoniae* in children. *Minerva Pediatr.* (2017) 69:156–60. 28178776 10.23736/S0026-4946.16.04866-0

[23] Brand PL Caudri D Eber E Gaillard EA Garcia-Marcos L Hedlin G Classification and pharmacological treatment of preschool wheezing: changes since. *Eur Respir J.* (2008) 2014:1172–7. 10.1183/09031936.00199913 24525447

[24] Ubukata K. *Mycoplasma pneumoniae*. *Nihon Yakurigaku Zasshi.* (2013) 141:287–9. 10.1254/fpj.141.287 23665560

[25] Kenri T Kawakita Y Kudo H Matsumoto U Mori S Furukawa Y Production and characterization of recombinant P1 adhesin essential for adhesion, gliding, and antigenic variation in the human pathogenic bacterium, *Mycoplasma pneumoniae*. *Biochem Biophys Res Commun.* (2019) 508:1050–5. 10.1016/j.bbrc.2018.11.132 30551878

[26] Kannan TR Musatovova O Balasubramanian S Cagle M Jordan JL Krunkosky TM *Mycoplasma pneumoniae* community acquired respiratory distress syndrome toxin expression reveals growth phase and infection-dependent regulation. *Mol Microbiol.* (2010) 76:1127–41. 10.1111/j.1365-2958.2010.07092.x 20199607 PMC2883071

[27] Rodriguez-Martinez CE Sossa-Briceño MP Castro-Rodriguez JA. Advantage of inhaled corticosteroids as additional therapy to systemic corticosteroids for pediatric acute asthma exacerbations: a cost-effectiveness analysis. *J Asthma.* (2020) 57:949–58. 10.1080/02770903.2019.1628254 31164017

[28] Pinto JM Wagle S Navallo LJ Petrova A. Risk factors and outcomes associated with antibiotic therapy in children hospitalized with asthma exacerbation. *J Pediatr Pharmacol Ther.* (2022) 27:366–72. 10.5863/1551-6776-27.4.366 35558351 PMC9088437

[29] Forno E Wang T Yan Q Brehm J Acosta-Perez E Colon-Semidey A A multiomics approach to identify genes associated with childhood asthma risk and morbidity. *Am J Respir Cell Mol Biol.* (2017) 57:439–47. 10.1165/rcmb.2017-0002OC 28574721 PMC5650086

[30] Benjaponpitak S Benjaponpitak A Kamchaisatian V Sasisakulporn C Santikul K Direkwattanachai C. Risk factors of relapse within eight weeks after an acute asthma exacerbation in Thai children. *J Med Assoc Thai.* (2002) 85 Suppl 4:S1041–8. 12549774

[31] Zhang B Jin R Guan RZ Lin RJ Chang DY Zhang LH Evaluation of the efficacy of Chinese children’s asthma action plan on the long-term management of children with asthma at home. *Zhonghua Yi Xue Za Zhi.* (2020) 100:3702–5. 10.3760/cma.j.ch112137-20200408-01125 33342148

[32] Sawanyawisuth K Chattakul P Khamsai S Boonsawat W Ladla A Chotmongkol V Role of inhaled corticosteroids for asthma exacerbation in children: an updated meta-analysis. *J Emerg Trauma Shock.* (2020) 13:161–6. 10.4103/JETS.JETS_116_19 33013097 PMC7472813

[33] Yeung TY Ewing C Malanowska A Zuberbuhler P Balcom M Liu J Home management of childhood asthma exacerbations. *Pulm Ther.* (2018) 4:149–57. 10.1007/s41030-018-0061-y 32026392 PMC6966973

